# Evaluation of the Radial Procurvatum Using the Center of Rotation of Angulation Methodology in Chondrodystrophic Dogs

**DOI:** 10.3389/fvets.2021.774993

**Published:** 2022-01-03

**Authors:** Minji Kwon, Danbee Kwon, Jonghyop Lee, Kichang Lee, Hakyoung Yoon

**Affiliations:** ^1^Department of Veterinary Medical Imaging, College of Veterinary Medicine, Jeonbuk National University, Iksan-si, South Korea; ^2^Bundang Leaders Animal Medical Center, Seongnam-si, South Korea; ^3^Nel Animal Medical Center, Anyang-si, South Korea

**Keywords:** canine, antebrachium, radius, joint orientation angle, radiograph

## Abstract

The radial joint orientation angles were calculated using the center of rotation of angulation (CORA) methodology within the frontal and sagittal planes in chondrodystrophic dog breeds, including Welsh Corgi, Dachshund, Pekinese, Poodle, Beagle and Maltese, and it was compared whether there is a statistically significant difference between the breeds. Radial joint orientation angles were obtained in eighty-eight dogs, including 23 Welsh Corgis, 16 Dachshunds, 14 Pekinese, 13 Maltese, 12 Poodles and 10 Beagles. Using the CORA methodology, the cranial proximal radial angle (CrPRA) and caudal distal radial angle (CdDRA) in the sagittal plane and medial proximal radial angle (MPRA) and lateral distal radial angle (LDRA) in the frontal plane were measured for the six breeds studied. The mean values of joint angles for each breed were compared statistically were observed. The CrPRA, CdDRA, and LDRA mean values of Dachshund and Welsh Corgi breeds were significantly smaller than other breeds, and in MPRA, Pekingese showed significantly smaller values than other breeds. This study confirms that the mean values of radial joint orientation angles can be significantly different among chondrodystrophic breeds. To accurately evaluate the degree of angular deformity of the radius, it may be helpful to refer to the average value for each breed with chondrodystrophy.

## Introduction

Angular limb deformities (ALD) can arise from congenital malformations, physeal injuries, nutritional imbalances, and other factors that can cause retardation of the growth plate (1–3). ALD affecting the radius and ulna are common in dogs (2). Retardation of growth of the distal ulnar physis (growth plate) results in a shortened ulna (4–7), which may lead to cranial bowing of the radius, valgus deformity at the carpal joint, and elbow subluxation (8, 9). In particular, chondrodystrophic breeds have impaired growth of the long bones, and often have a prematurely closed distal ulnar growth plate, resulting in angular deformities (10, 11).

The center of rotation of angulation (CORA) methodology of classifying the degree of angular deformity was adapted from human medicine and first applied to dogs in 2006. The degree and location of limb deformity can be assessed through CORA methodology (12). Measurement of joint orientation angles using CORA in patient with ALD may provide a guide to be used during corrective osteotomy (13–18).

In patients with unilateral ALD, the unaffected contralateral limb can be used as a surgical indicator, but it is difficult to provide appropriate values in patients with bilateral ALDs (19, 20). Therefore, it is necessary to study the joint reference angles in normal limbs to identify appropriate standards. While several previous studies have evaluated the joint orientation angles of canine long bones, research on the normal reference for each breed is insufficient. If inappropriate reference values are used, osteotomy for correction of angular deformity may result in iatrogenic translation or malalignment (13, 21). For this reason, it is particularly important that surgeons operating on patients with forelimb malformations have access to appropriate indicators.

The aim of this study was to calculate the radial joint orientation angles at the sagittal and frontal planes in several chondrodystrophic dog breeds, including Welsh Corgis, Dachshunds, Pekinese, Maltese, Poodles and Beagles, using the CORA methodology, and to compare the values from each breed.

## Materials and Methods

### Animals

This was the analysis of the radial joint orientation angles measured on orthogonal (medio-lateral and cranio-caudal) antebrachial radiographs in case series of chondrodystrophic dogs. Breeds such as Welsh Corgis, Dachshunds, Pekinese, Maltese, Poodles and Beagles were included in this study based on the classification of chondrodystrophic breeds found in the previous veterinary literature (22–27). For inclusion criteria, dogs with complete, normal and mature antebrachial skeletal bones were included. Dogs with fractures, osteoarthritis, tumors on radiographs, and those with clinical symptoms such as gait abnormality or pain in the extremities were excluded.

We conducted a search of the medical records at Jeonbuk National University, Bundang Leaders Animal Medical Center and Nel Animal Medical Center from January 2018 to June 2021. This study is a retrospective study in that it referred to radiographs already taken among chondrodystrophic breeds that visited from January 2018 to December 2019. This was a prospective study in that the forelimb radiography was additionally performed after obtaining the consent of the owner.

This study was approved by the Institutional Animal Care and Use Committee of Jeonbuk National University, Iksan-si, Jeollabuk-do, Republic of Korea (Approval No. JBNU 2021-040).

### Measurements

Digital images were assessed on a DICOM workstation, and conventional films were evaluated using radiographic view boxes. All evaluations were performed on the sagittal and frontal planes of the radiographic images. Measurements from digital images were taken using electronic calipers and measurements from conventional radiographs were taken using manual calipers. Radiographs were reviewed in random order by independent observers (M.K. and H.Y).

**Figure 1:**
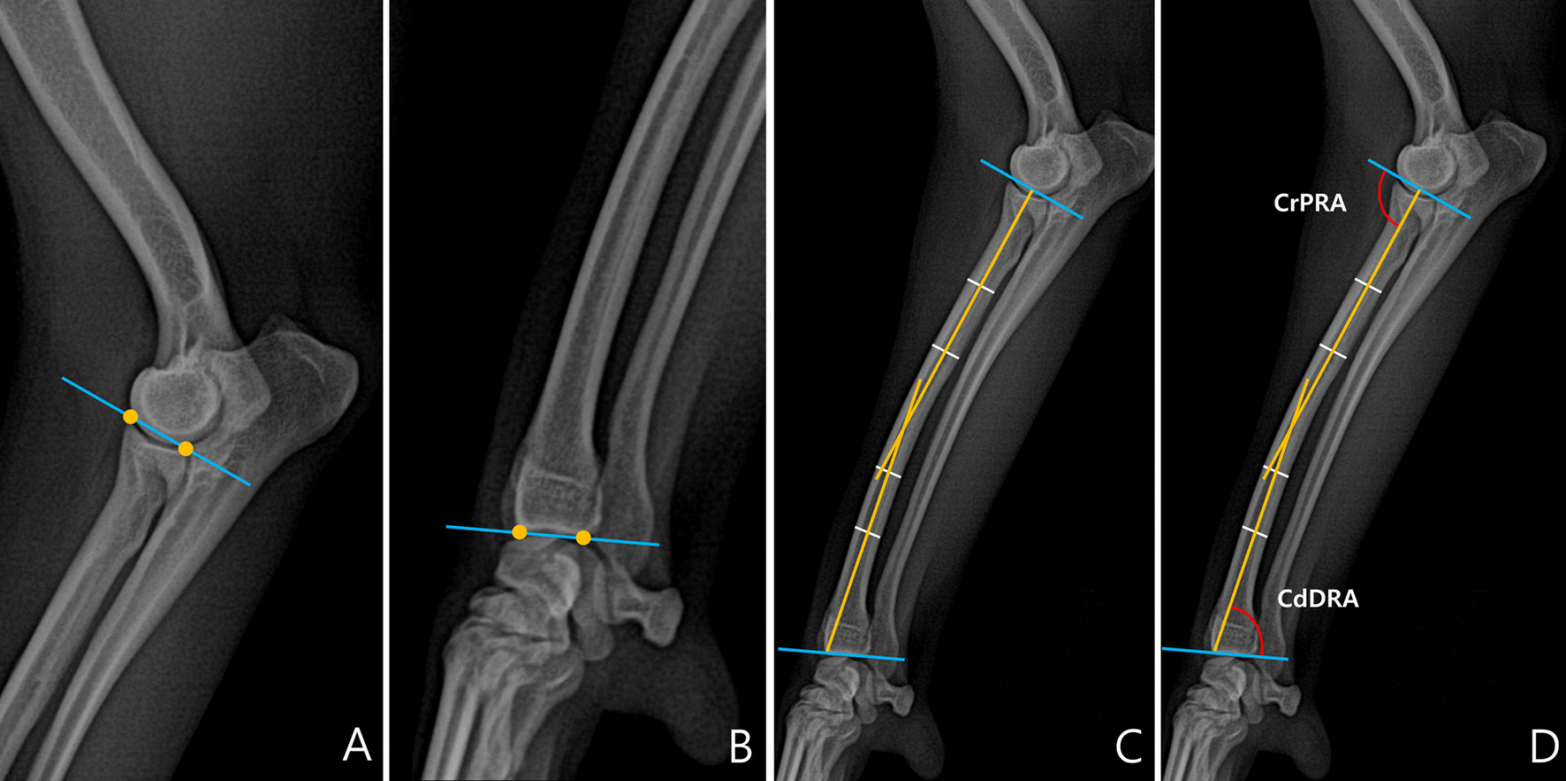
Normal elbow joint orientation angle, sagittal plane. **(A)** To draw the joint orientation line head in the proximal part of the radius, connect two points from the cranial aspect to the caudal aspect of the radial head in the proximal part of the radius. **(B)** To draw the joint orientation line in the distal part of the radius, connect two points from the cranial aspect to the caudal aspect of the radial articular face from the distal part of the radius. **(C)** To draw the radial anatomic axis, connect two mid-diaphyseal points each for proximal and distal radial segments. **(D)** Cranial proximal radial angle (CrPRA) measured between the proximal radial anatomic axis and the elbow joint orientation line. Caudal distal radial angle (CdDRA) measured between the distal radial anatomic axis and carpal joint orientation line.

**Figure 2:**
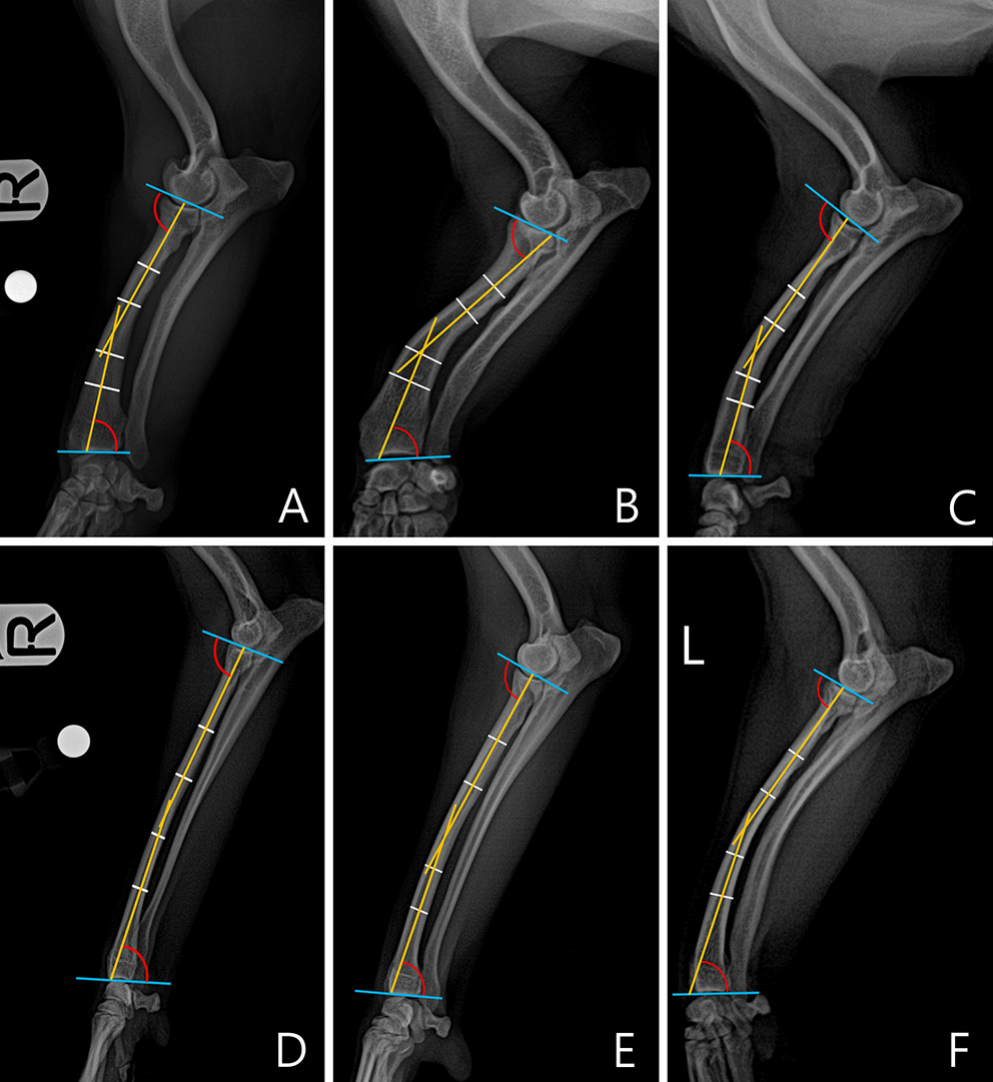
An example of a measured cranial proximal radial angle (CrPRA) and caudal distal radial angle (CdDRA) for each breed. The subjects images here were randomly selected. **(A)** Welsh Corgi. **(B)** Dachshund. **(C)** Pekingese. **(D)** Poodle. **(E)** Beagle. **(F)** Maltese.

To measure joint orientation angles, we referred to a previous study that had evaluated radial deformity using CORA (13). Joint orientation angles were measured in the sagittal and frontal planes. First, we obtained the joint orientation lines at the proximal and distal parts of the radius (Figures 1A,B). Since the radius of dogs naturally curves to cranially, the radial anatomic axes were drawn as the proximal diaphysis and the distal diaphysis (Figure 1C). By measuring the angles between the joint orientation lines and the anatomic axes for both elbow and carpus, the cranial proximal radial angle (CrPRA) and caudal distal radial angle (CdDRA) were obtained (Figure 1D). An example of the measurement of the joint orientation angles in the sagittal plane for each breed is shown in Figure 2. The subjects used in the example were randomly selected.

**Figure 3:**
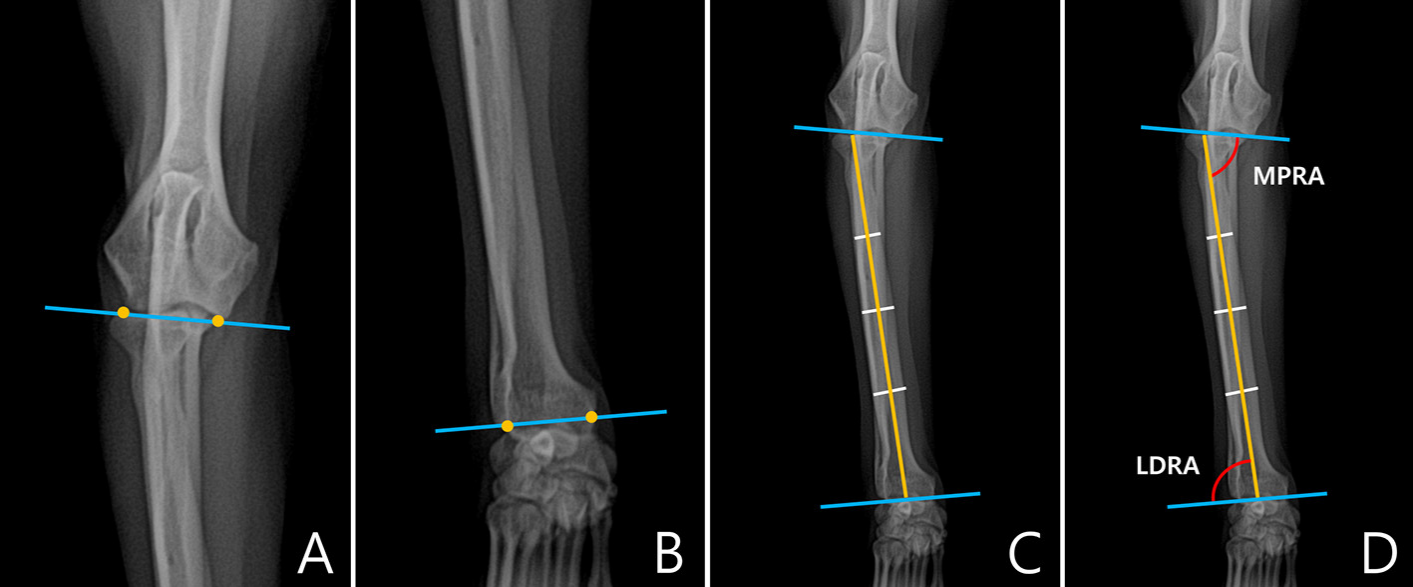
Normal elbow joint orientation angle, frontal plane. **(A)** To draw the joint orientation line in the proximal part of the radius, connect two points from the lateral aspect of the radial head to the medial coronoid process or from the distal-most aspect of the humeral condylar capitulum to the trochlea in the elbow. **(B)** To draw the radial anatomic axes in the distal part of the radius, connect two points from the lateral aspect of the radial articular face to the most medial aspect of the radial articular face ignoring styloid process were determined. **(C)** To draw the radial anatomic axis, connect three mid-diaphyseal points at 25, 50, and 75% along the length of the radius. **(D)** Medial proximal radial angle (MPRA) measured between the proximal radial anatomic axis and the elbow joint line. Lateral distal radial angle (LDRA) measured between the distal radial anatomic axis and the carpal joint line.

**Figure 4:**
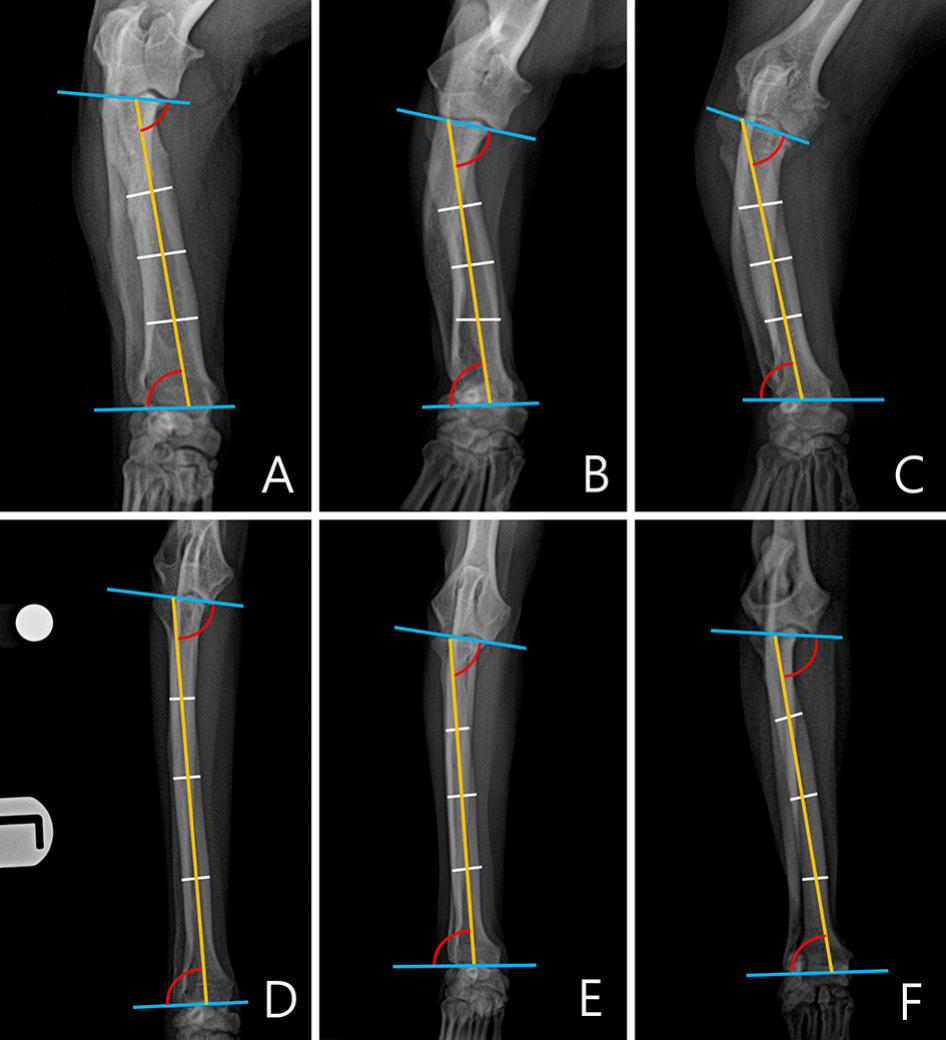
An example of a measured medial proximal radial angle (MPRA) and lateral distal radial angle (LDRA) for each breed. The subjects images here were randomly selected. **(A)** Welsh Corgi. **(B)** Dachshund. **(C)** Pekingese. **(D)** Poodle. **(E)** Beagle. **(F)** Maltese.

In the frontal plane, joint orientation angles were obtained in a similar way to the sagittal plane. The joint orientation lines were drawn at the proximal and distal parts of radius (Figures 3A,B). To obtain the anatomic axis, a line perpendicular to the diaphysis was drawn at 25, 50, and 75% of the length of the radial diaphysis, and the center points of the lines were connected. When the midpoints of the three parts did not match, the line connecting the midpoints of 25 and 75% of the two parts was defined as the anatomic axis (Figure 3C). Next, joint orientation angles for both the elbow and carpus were measured, and the medial proximal radial angle (MPRA) and the lateral distal radial angle (LDRA) measurements were obtained (Figure 3D). An example of the measurement of the joint orientation angles in the frontal plane for each breed is shown in Figure 4. The subjects used in the example were randomly selected.

### Statistics

All statistical tests were selected and conducted by one of the authors (M.K.). All angles were analyzed using SPSS software, and the mean and standard deviation values were calculated for each breed. Breeds were compared through a one-way ANOVA and *post-hoc* Scheffe's test. All experimental values were evaluated as significant when the *p*-value was < 0.05.

## Results

The 88 dogs representing six breeds were selected for the study. The chondrodystrophy group included Welsh Corgis (*n* = 23), Dachshunds (*n* = 16), Pekinese (*n* = 14), Maltese (*n* = 13), Poodles (*n* = 12) and Beagles (*n* = 10). Mean age was (6.88 ± 4.18 years), and mean body weight was (7.56 ± 3.92 kg). A total of 43 males (36 castrated) and 45 females (30 spayed) were selected (Table 1).

**Table 1:** Demographics and gonadal status in each group.

**Breed**	**Age (years)**	**BW (kg)**	**Gender**				***N***
			**MI**	**MC**	**FI**	**FS**	
Welsh Corgi	5.48 ± 4.21	13.14 ± 2.61	2	11	3	7	23
Dachshund	8.63 ± 4.24	7.72 ± 2.61	1	10	1	4	16
Pekingese	9.54 ± 4.63	5.63 ± 1.28	0	3	4	7	14
Maltese	6.77 ± 2.86	4.41 ± 1.76	1	8	0	4	13
Poodle	6.08 ± 3.32	5.07 ± 1.32	1	4	3	4	12
Beagle	4.48 ± 3.82	9.38 ± 1.56	2	0	4	4	10

*Age and body weight are expressed as mean ± standard deviation (SD) values. BW, body weight; MI, male intact; MC, male castrated; FI, female intact; FS, female spayed; N, total number of each group*.

The mean ± standard deviation (SD) values of CrPRA, CdDRA, MPRA, and LDRA for each breed are summarized in Table 2.

**Table 2:** The mean ± standard deviation (SD) values of the CrPRA, CdDRA, MPRA, and LDRA for Welsh Corgis, Dachshunds, Pekinese, Maltese, Poodles and Beagles.

**Breed**	**CrPRA**	**CdDRA**	**MPRA**	**LDRA**
Welsh Corgi	77.62 ± 5.09	59.90 ± 4.44	70.53 ± 5.19	77.63 ± 4.12
Dachshund	75.69 ± 4.56	64.79 ± 4.43	70.96 ± 4.49	76.69 ± 5.37
Pekingese	86.52 ± 4.13	66.53 ± 3.59	61.97 ± 5.30	82.95 ± 3.59
Maltese	86.12 ± 4.65	77.59 ± 4.72	73.51 ± 4.83	87.24 ± 5.81
Poodle	88.13 ± 3.91	71.11 ± 3.80	77.91 ± 3.44	89.60 ± 2.03
Beagle	84.39 ± 4.30	75.62 ± 3.87	74.82 ± 1.87	86.98 ± 1.58

*CdDRA, caudal distal radial angle; CrPRA, cranial proximal radial angle; LDRA, lateral distal radial angle; MPRA, medial proximal radial angle*.

**Figure 5:**
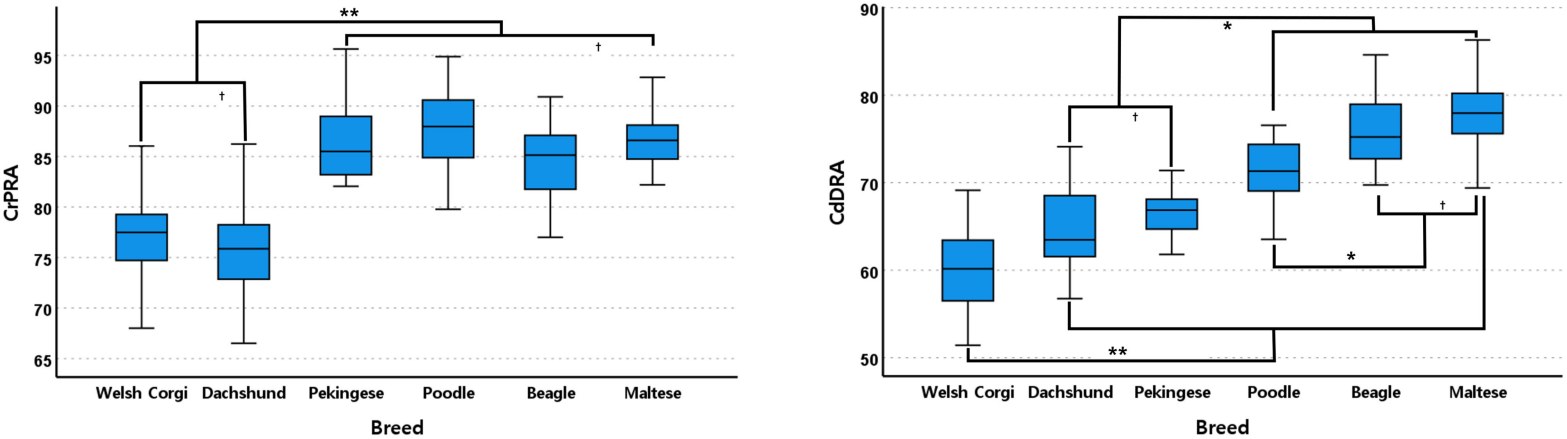
Box and whisker plots of cranial proximal radial angle (CrPRA) and caudal distal radial angle (CdDRA) of Welsh Corgis, Dachshunds, Pekinese, Poodles, Beagles and Maltese. Boxes indicate sample variation and whiskers represent standard deviation. “*” Indicates statistically significant difference (*P* < 0.05) and “†” indicates statistically no significant difference (*P* ≥ 0.05), determined by analysis of variance followed by Scheffe's *post-hoc* analysis. *P < 0.05, **P < 0.001.

In the sagittal plane, Dachshunds and Welsh Corgis had significantly lower CrPRA mean values than other breeds (*P* < 0.001, Figure 5). There were no significant differences between Pekingese, Beagles, Poodles, and Maltese breeds.

Welsh Corgis had the lowest CdDRA mean value and had a significantly lower angle value than other breeds (*P* < 0.001, Figure 5). Dachshunds and Pekingese had the second lowest CdDRA mean value and a significantly lower angle value than those of Maltese, Poodles, and Beagles (*P* ≤ 0.020, Figure 5). In fourth order, Poodles had smaller CdDRA mean values and were significantly smaller than Maltese and Beagles (*P* ≤ 0.039, Figure 5).

**Figure 6:**
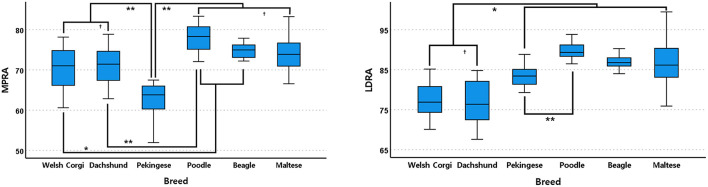
Box and whisker plots of medial proximal radial angle (MPRA) and lateral distal radial angle (LDRA) of Welsh Corgis, Dachshunds, Pekinese, Poodles, Beagles and Maltese. Boxes indicate sample variation and whiskers represent standard deviation. “*” Indicates statistically significant difference (*P* < 0.05) and “†” indicates statistically no significant difference (*P* ≥ 0.05), determined by analysis of variance followed by Scheffe's *post-hoc* analysis. *P < 0.05, **P < 0.001.

In the frontal plane, Pekingese had the lowest MPRA mean value and was significantly different from other breeds (<0.001, Figure 6). In the next order, Welsh Corgis had a smaller value, and it was statistically different from Beagles and Poodles (*P* ≤ 0.041, Figure 6). Dachshunds had the third smallest MPRA mean value which was statistically different from Poodles (*P* < 0.001, Figure 6). There was no significant difference between the Maltese, Beagle and Poodle breeds.

In the LDRA mean value, Dachshunds and Welsh Corgis showed the lowest values (*P* ≤ 0.003, Figure 6). Pekingese had the second lowest LDRA mean value and a significantly lower angle value than those of Poodles (*P* < 0.001, Figure 6). There was no significant difference between the remaining breeds, the Maltese, Beagle and Poodle breeds.

The differences in the CrPRA, CdDRA, MPRA, and LDRA measurements between male and female dog were not statistically significant.

## Discussion

In this study, we present the average joint orientation angle values for several dog breeds and demonstrate that there are significant differences in these measurements even though all breeds analyzed are classified as chondrodystrophic. A previous study on the joint orientation angle of the radius in dogs showed that there was no distinct difference between chondrodystrophy and non-chondrodystrophy categories (2). However, the study did not differentiate between diverse breeds, such as Welsh Corgis, Dachshunds, Beagles, and Maltese, within the chondrodystrophy category. So, it is possible that any differences between the breeds were not reflected in the study. Our study confirms that there were significant differences of joint orientation angles among several breeds even within the category of chondrodystrophic breeds and presents the average value for each breed. The study conducted by Knapp compared the degree of radial procurvatum between chondrodystrophic and non-chondrodystrophic dogs, referencing the radial joint orientation angle values previously established for Labrador Retriever and non-Labrador Retriever limbs by Fasanella (28). Comparing the mean values of the radial joint orientation angle measured by Fasanella with the mean values of the present study, chondrodystrophic breeds were smaller than Labrador Retriever and non-Labrador Retriever dogs in most items except for the CdDRA mean value of Maltese and the LDRA mean value of Maltese, Poodle, and Beagle breeds.

The growth of the antebrachium is complex, in that the radius and ulna must develop in a synchronized manner (2). However, when there is discrepancy in the growth rate of the ulna and radius, radial curvature may occur (29, 30). This ALD results in joint incongruity and uneven load distribution in the elbow and carpus, which can cause secondary osteoarthritis and lameness (31).

The chondrodystrophic breeds, in which ALD is relatively common, have short legs and a similar trait to disproportionate dwarfism in humans (6). The characteristic of short legs associated with chondrodystrophy is related to a fibroblast growth factor 4 retrogene (FGF4RG) (10). Expression of FGF4RG causes over-activation of fibroblast growth factor receptor factor 3 (FGFR3) (10, 32, 33). In a similar way, several mutations that cause excessive activation of FGFR3 can cause achondroplasia in humans (34, 35). Enhanced activity of FGFR3 in mice also causes disruption of growth plate structures and enhances terminal chondrocyte differentiation (36).

According to one study, the allele frequency of FGF4RG was variably detected among different breeds of chondrodystrophy (37). The cause of chondrodystrophy was considered to be the presence of two FGF4RG, one on chromosome 12 (12-FGF4RG) and one on chromosome 18 (18-FGF4RG) (10, 32, 37). The most disproportionate form of dwarfism was found in breeds with both FGF4RGs, such as Dachshunds, Corgis, and Basset Hounds, supporting the idea that both FGF4RGs affect long-bone length (32, 37, 38). Other breeds, including Pekingese, Beagle, Poodle, and Maltese, had relatively low levels of FGF4RG frequency (32, 37). In this study, Welsh Corgis and Dachshunds had the smallest values in CrPRA, CdDRA, and LDRA, and the second smallest values in MPRA, suggesting that the short leg length caused by the expression of FGF4RG induced a relatively small joint orientation angle. Similarly, in cats with disproportionate dwarfism, the joint orientation angles measured using the CORA methodology were confirmed to be smaller than those of normal cats (39). In addition, in this study, Pekingese showed the smallest values in MPRA compared with breeds, and the third smallest CdDRA and LDRA mean values after Dachshund and Welsh Corgi. While both Welsh Corgis and Dachshunds had high 12-FGF4RG and 18-FGF4RG frequencies, Pekingese had medium 12-FGF4RG and high 18-FGF4RG frequencies (37). Our results show that it may be necessary to study to what extent each of the two FGF4RGs affects long-bone length, and how they interact with each other.

Surgical goals for ALD correction of forelimbs in skeletal mature dogs include re-establishment of radial misalignment, length mismatch, and elbow incongruity (13). Surgical correction of ALD can prevent osteoarthritis progression and improve joint function (40). Various osteotomies for correcting ALD of the antebrachial deformities, including dome osteotomy, have been previously proposed (13), and in order to successfully realign the forelimb through such osteotomies, a normal range of joint orientation angle of each breed is required. By applying an accurate normal joint orientation angle range for each breed, it is possible to more accurately evaluate the degree of deformity (41).

There were several limitations to this study. First, due to the limitation of a retrospective study, the imaging acquisition conditions were not the same for all images, and the X-ray beam center could not be matched to the joint to be measured. And since the process of measuring the angle in the radiographic image was also conducted by two observers (M.K. and H.Y.), a limitation is that of interobserver variability. Other limitations include the small sample size and lack of breed diversity. And since the evaluation of joint orientation angle in this study did not reflect the caudal angulation of radial midshaft, the overall caudal angulation of the radius is still unclear. Also when drawing the anatomic axis in the frontal plane of radiographs of the radius, further studies are needed on cases where the midpoints of 25, 50, and 75% of the length of the radial diaphysis do not fit. Orthopedic examinations were performed only through physical and radiographic examinations but not based on high-quality images, such as CT images and three-dimensional geometry of canine radius by computer-aided design software. ALD evaluation through orthogonal radiography has a limitation in that the accuracy of measurement may be lowered if there is a torsional component excesses 15°, resulting in radiographic errors > 5° in the frontal plane (42). Since the degree of torsion in this study was subjectively evaluated through orthogonal radiographs, the limitation is that the three-dimensional degree of torsion was not considered. Therefore, the mean joint angle values for each chondrodystrophic breed presented through this study can be regarded as preliminary values.

In conclusion, the results of this study confirm significant differences among chondrodystrophic breeds and presents the average values for each breed. Accordingly, average values of the radial joint orientation angles appropriate to each breed should be applied when evaluating the angular deformities affecting the radius. To the best of our knowledge, this is the first study to present the average valves of radial joint orientation angles and the difference among chondrodystrophic dogs.

## Data Availability Statement

The original contributions presented in the study are included in the article/supplementary materials, further inquiries can be directed to the corresponding authors.

## Ethics Statement

The animal study was reviewed and approved by Institutional Animal Care and Use Committee. Written informed consent was obtained from the owners for the participation of their animals in this study.

## Author Contributions

MK and HY: conception, design, and drafting. MK, DK, JL, and HY: acquisition of data. MK, HY, DK, JL, and KL: analysis, interpretation of data, revision for intellectual content, and final approval of the completed article. All authors contributed to the article and approved the submitted version.

## Funding

This work was supported by the National Research Foundation of Korea and funded by a grant from the Korean government (No. 2021R1C1C1006794).

## Conflict of Interest

The authors declare that the research was conducted in the absence of any commercial or financial relationships that could be construed as a potential conflict of interest.

## Publisher's Note

All claims expressed in this article are solely those of the authors and do not necessarily represent those of their affiliated organizations, or those of the publisher, the editors and the reviewers. Any product that may be evaluated in this article, or claim that may be made by its manufacturer, is not guaranteed or endorsed by the publisher.

## Abbreviations

CORA: center of rotation of angulation
CrPRA: cranial proximal radial angle
CdDRA: caudal distal radial angle
MPRA: medial proximal radial angle
LDRA: lateral distal radial angle
ALD: angular limb deformities
FGF4RG: fibroblast growth factor 4 retrogene
FGFR3: fibroblast growth factor receptor factor 3.
